# Functional cognitive performance augments cognitive screening data in older adults

**DOI:** 10.3389/fnagi.2025.1535146

**Published:** 2025-06-19

**Authors:** Timothy S. Marks, Gordon Muir Giles, Dorothy Farrar Edwards

**Affiliations:** ^1^Department of Occupational Therapy, University of Missouri, Columbia, MO, United States; ^2^Department of Occupational Therapy, Samuel Merritt University, Oakland, CA, United States; ^3^Departments of Kinesiology and Medicine, University of Wisconsin-Madison, Madison, WI, United States

**Keywords:** IADL, functional cognition, cognition, assessment, performance-based testing

## Abstract

**Background:**

Understanding the association of older adults’ cognitive ability with performance of instrumental activities of daily living (IADL) is critical to identifying their community health care support needs. We compared differences in performance-based IADL assessment scores among older adults according to their cognitive ability as measured by the Montreal Cognitive Assessment (MoCA).

**Methods:**

Using data from a larger study we performed a cross-sectional analysis of 259 community-dwelling adults aged 55–93 years. Participants were categorized into one of three groups based on their MoCA score: mildly impaired (19–22), borderline (23–25), or unimpaired (26–30). The Performance Assessment of Self-care Skills Checkbook Balancing and Shopping Task (PCST) and the Weekly Calendar Planning Activity 17-item version (WCPA-17) were used to assess IADL. A MANCOVA analyzed the effect of MoCA group on the performance-based IADL assessments while controlling for education.

**Results:**

The MANCOVA was statistically significant, *F*(4, 508) = 16.445, *p* < 0.001; Wilks’ λ = 0.784; η_*p*_^2^ = 0.115. Follow-up univariate ANCOVAs showed that PCST Total Cues adjusted mean score [*F*(2, 255) = 20.006, *p* < 0.001; η_*p*_^2^ = 0.136] and WCPA-17 Accuracy adjusted mean scores [*F*(2, 255) = 23.216, *p* < 0.001; η_*p*_^2^ = 0.154] were significantly different among MoCA groups, with medium-large effect sizes.

**Conclusion:**

The tripartite group categorization of the MoCA largely parallels ability on two independent performance-based IADL assessments, a subset of individuals borderline or unimpaired on the MoCA had difficulties with complex IADL identified by performance-based IADL assessments indicating comprehensive evaluations of older adults would benefit from including both types of assessments.

## Introduction

Medication management, shopping, meal preparation, and financial management are all instrumental activities of daily living (IADL) necessary for independent community living (Lawton and Brody, 1969). These everyday activities require the integration of executive functions, motor skills, and a range of other cognitive abilities that may all become less efficient with increasing age (Lezak, 1982; Royall et al., 2007; Tucker-Drob, 2011). Community living skills have been hypothesized to be supported by multiple domains of cognitive abilities, as a result, reliable assessment of the cognitive capacity to perform IADL in older adults has proved difficult (Giovannetti et al., 2023). However, IADL metrics have been shown to be predictive of cognitive decline, further loss of IADL independence, increased risk of hospital admission, and increased risk of 30 days readmission after hospital discharge (Lau et al., 2015; Brown et al., 2019; Schiltz et al., 2020; Cloutier et al., 2021) all of which underline the importance of accurate assessment of the cognitive skills essential for IADL performance. Thus, understanding that cognitive ability is associated with IADL performance is of particular importance to healthcare practitioners who work with patients to promote successful aging, quality of life, and community independence.

Functional cognitive assessment is an approach to standardized IADL measurement that uses performance-based testing within the context of simulated real-life activities (Giles et al., 2020). Functional cognition is a multidimensional construct theorized to be associated with successful performance of IADL and independent community living (Wesson and Giles, 2019) and examines how a person marshals all their abilities during goal directed functional activities. Functional cognitive assessments are designed to challenge the cognitive functions needed for managing everyday life and provide clinically relevant information that can be used to inform intervention selection (Barco et al., 2019). These assessments examine how an individual approaches different functional tasks by allowing the use of compensatory strategies. Observation of test performance provides clinical insight into an individual’s functional-cognitive performance capacity in everyday life (Bar-Haim Erez and Katz, 2018). For example, the Performance Assessment of Self-care Skills (PASS; Rogers et al., 2016) and the Executive Function Performance Test (EFPT; Baum et al., 2008) which are sensitive to mild cognitive impairment (MCI; Rodakowski et al., 2014) summarize the number and type of cues needed to complete simulated daily living tasks; the Weekly Calendar Planning Activity (WCPA; Toglia, 2015) profiles overall accuracy of scheduling appointments into a weekly calendar. The performance outcomes provide clinicians with a different conceptualization and more distinct information over and above that which is provided by cognitive screening and assessment. Such information includes the individual’s capacity to plan, self-initiate, sequence and terminate a complex activity, and may also assess the test takers self-awareness of their performance in real time. Clinicians rely on functional cognitive assessment to accurately predict everyday function, safety, and to provide clinical recommendations and intervention as indicated (American Occupational and Therapy Association, 2021).

Although some individuals with mild cognitive impairment may have difficulty performing complex everyday tasks, the associated decline in activity performance is poorly characterized by available systems of measurement (Cloutier et al., 2021; Corbo and Casagrande, 2022). Despite this major limitation, increased difficulty with everyday activity performance and inabilities to compensate can have direct implications for an individual’s safety and community independence and can lead to further emotional and functional decline (De Vriendt et al., 2012). Unimpaired performance of IADLs was initially a prerequisite for the diagnosis of mild neurocognitive disorder, however, current evidence suggests that unrecognized deficits in complex IADL, also termed “preclinical” disability, may presage the need for ongoing monitoring and support (Lindbergh et al., 2016; Fieo and Stern, 2018; Lee et al., 2019). Functional cognitive assessments could facilitate early detection of these functional difficulties with complex IADL and can indicate avenues to increase intervention and support (Jekel et al., 2015; Cloutier et al., 2021). A growing population of older adults are predicted to experience cognitive decline rendering functional cognitive assessments critical to understanding the complex interplay between cognition and everyday life competencies (Prince et al., 2013). Thus, there is a need to examine how subtle differences in cognitive abilities operationalize to functional cognitive assessment outcomes to further understand the link between these two types of assessment and their relationship to illness progression.

The main objective of this paper is to compare functional cognitive performance among community-dwelling older adults according to their cognitive ability using an extensively validated cognitive screening measure, the Montreal Cognitive Assessment (MOCA; Nasreddine et al., 2005). The MoCA is the most widely used screening test of cognition by occupational therapy practitioners who are frequently called upon to make recommendations regarding an individual’s need for community support (Manee et al., 2020). Screening tests typically include a cut-off value used to group individuals into categories of “unimpaired” or “impaired” which are then used to make further decisions regarding assessment. The original criterion for impairment on the MoCA (cut-off of 26) resulted in high rates of false-positive classifications in multiple studies (Luis et al., 2009; Rossetti et al., 2011; Davis et al., 2015; Elkana et al., 2020). Although there is no consensus regarding an optimal cutoff score, a meta-analysis of MoCA validation studies using strict criteria to define MCI found that a MoCA cut-off of 23 provided more accurate classification across a variety of demographic parameters (Carson et al., 2018). To further minimize misclassification and potential loss of information from a binary classification approach, a number of researchers recommend the inclusion of a transitional or “indecisive” area (individuals neither definitively unimpaired nor impaired) by using two separate cut-off points (Brenkel et al., 2017; Thomann et al., 2020; Yang et al., 2021; Völter et al., 2023). Following this multiple cut-off approach on the MoCA, we categorized individuals with MoCA scores of 19–22 as mildly impaired, those with scores between 23 and 25 as borderline impaired, and those with scores of 26 or above as unimpaired. Individuals scoring below 19 on the MoCA were considered as evidencing greater than mild impairments and were not included in this study. We hypothesized that performance on the functional cognitive assessments would have a linear relationship with scores on the MoCA and further would be related to the sensitive tripartite grouping of MoCA scores with higher scores on the MoCA reflecting greater independence on the functional cognitive assessments. Further we hypothesized that the MoCA groups would correspond with significantly different scores on each functional cognitive assessment.

## Materials and methods

### Research design

This cross-sectional study uses data gathered as part of the Menu Task validation study (Al-Heizan et al., 2020; Marks et al., 2020, 2021a,2021b).

### Participants and recruitment

Data collected for the primary study and analyzed here were obtained from a convenience sample of 287 community-dwelling adults recruited in Madison, Wisconsin, and the surrounding area. We retained 259 participants for this analysis removing those who did not complete either the PCST or WCPA-17 and those with MoCA scores below 19. Inclusion criteria were age 55 years or older, living independently in the community (i.e., not in assisted living, skilled nursing facility, or other institutionalized settings, and who reported that they were independent in community-living skills), willingness and ability to read and write in English, and vision, hearing, and motor skills adequate for testing.

The study was approved by the University of Wisconsin – Madison Institutional Review Board. All participants provided written informed consent prior to participation, and the study protocol complied with the Declaration of Helsinki.

### Measures

#### Cognition screening test

##### Montreal Cognitive Assessment (MoCA)

The MoCA (Nasreddine et al., 2005) is a multi-component cognitive screening test that takes approximately 10 min to administer. The test evaluates seven domains of cognition including visuospatial/executive function, naming, memory, attention, language, abstraction, delayed recall and orientation. Possible total scores range from 0 to 30 with higher scores indicating better performance and with a one-point adjustment for individuals with ≤ 12 years of education.

### Performance-based functional cognition measures

#### Performance Assessment of Self-care Skills (PASS) Checkbook Balancing and Shopping task (PCST)

The PASS (Rogers et al., 2016) includes 26 subtests that measure activities of daily living (ADL) and IADL skills and is intended to assist clinicians in planning interventions. Of the available subtests 14 are described as having a cognitive emphasis (C-IADL). The PASS Checkbook Balancing and Shopping tasks (PCST) were used for this study as together these two subtests have been found to be as sensitive in discriminating between individuals with MCI and healthy older adults as the combined 14 C-IADL subtests (Rodakowski et al., 2014). The Checkbook Balancing task requires test-takers to pay bills by writing checks and correctly balance a checkbook ledger. The Shopping task requires test-takers to identify and select grocery items, handle money, and use discount coupons to make purchases. The PASS uses a cueing hierarchy that includes nine distinct levels in which increasingly directive cues are provided by the test administrator until the test taker correctly completes the task. Providing three cues at a specific level of assistance is indicative of the need to provide a higher level cue, though the test administrator is to use clinical judgment to ultimately determine when a higher level cue is needed. PCST scores are based on the combined number of cues required for independence and adequacy (quality) on each task (i.e., Total Cues), with lower numbers indicating better performance. PCST Total Cues scores start at 0 (no cues needed), and there is no predefined maximum although the number of cues is constrained by task completion. The PCST can be administered in approximately 15–20 min (Rogers et al., 2016).

#### 17-item Weekly Calendar Planning Activity (WCPA-17)

The WCPA is a performance-based assessment that examines how deficits in functional cognition affect a person’s ability to perform the complex activity of entering appointments into a weekly calendar (Toglia, 2015). The test administrator outlines the rules and requirements of the test and offers an opportunity to ask clarifying questions. The test-taker is instructed that no further verbal interaction is permitted. No cueing or assistance is provided by the test administrator during the test; however, the test-taker must ignore questions that attempt to distract them from the task. The 17-item version (WCPA-17) used for this study requires scheduling 17 randomly ordered appointments into a 1 week calendar while minimizing errors (e.g., repetition errors, location errors, time errors) managing potential scheduling conflicts, and adhering to five pre-specified rules. Multiple scores are derived from the WCPA-17, including Accuracy (the number of appointments entered without errors), Rules, Appointments Entered, Strategies, Planning time, Total time, and Efficiency (number of accurate appointments/time). Higher scores on Accuracy, Appointments Entered, Strategies, and Rules indicate better performance. A lower Efficiency score indicates better performance. The WCPA-17 is widely used with older adults for whom normative values have been published (Toglia, 2015). Accuracy score was used to represent the WCPA-17 because it is the primary indicator of overall task competency. The Accuracy score sums the number of entered appointments without errors. Scores on the WCPA-17 range from 0 to 17. The WCPA-17 can be administered in approximately 15–30 min (Toglia, 2015).

### Study procedures

Testing occurred per participant convenience at community settings or at the Occupational Therapy Department at the University of Wisconsin – Madison. Testing was completed by trained occupational therapy graduate students who met participants in office-like distraction-free environments. Locations included reserved study rooms in libraries, private offices in community centers, private spaces in retirement communities. Demographic information including age, sex, race, education (in years), and the number of self-reported chronic health conditions were collected from participants. All data were collected in one study visit which lasted 70–90 min in which participants were offered breaks. Participants received remuneration of $25 in cash.

### Analysis

Descriptive statistics were computed for continuous variables and frequency distributions for categorical variables. All variables were inspected for normality to guide analysis. Total sample mean values were used to replace three values of isolated, missing demographic information. The number of chronic health conditions and the PCST Total Cues score were log transformed to reduce right skew. The transformations resulted in normal distributions for the variables.

Demographic variables were examined to assess whether linear relationships existed between them and PCST Total Cues scores and WCPA-17 Accuracy scores. When a correlation of 0.30 or higher (Portney, 2020) was present the influence of such demographic variables was controlled for in the subsequent analyses.

To examine the relationship between the MoCA score (as a continuous variable, 19–30) and performance on the PCST Total Cues scores and WCPA-17 Accuracy scores, we performed two separate linear regression analyses.

In order to meet the main objective of this study, each participant was assigned to one of three groups based on their performance on the MoCA. The first group was comprised of individuals with MoCA scores categorized as mildly impaired (scores 19–22). The second group was comprised of individuals with MoCA scores categorized as borderline (scores 23–25). The third group was comprised of individuals with MoCA scores categorized as unimpaired (scores 26–30).

One-way analyses of variance (ANOVA) and chi-squared analyses were used to examine differences in demographic characteristics among the three MoCA groups.

A multivariate analysis of covariance (MANCOVA) was conducted to analyze the effect of cognitive ability defined by MoCA group (independent variable) on measures of functional cognitive performance (dependent variables). Follow-up ANCOVAs and *post hoc* tests were performed.

Bonferroni corrections were used for multiple comparisons with *p*-values set at 0.008 to indicate significance (0.05/6 = 0.008). Estimates of effect sizes were quantified via partial eta squared to determine the magnitude of significant group differences and were interpreted as: 0.01 = small, 0.06 = medium, and 0.14 = large (Cohen, 1988). SPSS version 27 was used for all analyses (IBM Corp., Armonk, NY).

## Results

The mean age of participants was 69.5 (SD = 8.0) years, and the sample was predominantly female (74.9%) and White (83.8%). On average, the participants were college educated, with a mean 15.7 (SD = 3.3) years of education. The sample was relatively healthy with a mean number of self-reported chronic health conditions of 1.0 (SD = 1.1; see Table 1). An educational correction was added to the MoCA score for 23.2% of the sample who had 12 or fewer years of education. The overall MoCA mean score was 24.8 (SD = 2.9; see Table 1).

**TABLE 1:** Demographic characteristics and scores on study measures.

Variable	Full sample *N* = 259	MoCA unimpaired (*n* = 114)	MoCA borderline (*n* = 82)	MoCA mildly impaired (*n* = 63)	*p* =
	M (SD)	M (SD)	M (SD)	M (SD)	
Age	69.5 (8.0)	67.7 (6.4)	71.4 (9.1)	70.3 (8.4)	0.003
Chronic health conditions	1.0 (1.1)	0.9 (1.1)	1.0 (1.1)	1.1 (1.1)	0.271
Education	15.7 (3.3)	16.7 (3.3)	15.6 (3.4)	14.0 (2.3)	< 0.001
	***n* (%)**	***n* (%)**	***n* (%)**	***n* (%)**	
Sex					0.371
Female	194 (74.9)	90 (79.0)	60 (73.2)	44 (69.8)	–
Male	65 (25.1)	24 (21.1)	22 (26.8)	19 (30.2)	–
Race*^a^*					–
White	217 (83.8)	102 (89.5)	72 (87.8)	43 (68.3)	–
Black	29 (11.2)	6 (5.3)	7 (8.5)	16 (25.4)	–
Other	13 (5.0)	6 (5.3)	3 (3.7)	4 (6.4)	–
MoCA	24.8 (2.9)	–	–	–	–
PCST Total Cues	8.5 (7.5)	–	–	–	–
WCPA-17 Accuracy	11.0 (4.1)	–	–	–	–

^a^Insufficient group representation to run χ^2^ as either full or collapsed factor variable. MoCA, Montreal Cognitive Assessment, possible range 0–30; PCST, Performance Assessment of Self-care Skills Checkbook Balancing and Shopping Task, possible range 0+; WCPA-17, Weekly Calendar Planning Activity 17-item version, possible range 0–17.

Of the demographic variables, only education had correlations above 0.30 with the PCST Total Cues score and WCPA-17 Accuracy score. Two separate linear regression analyses were performed controlling for education. The overall model predicting PCST Total Cues scores that included MoCA scores and education (years) as continuous independent variables was statistically significant [*F*(2, 256) = 36.25, *p* < 0.001, adjusted *R*^2^ = 0.22]. The overall model predicting WCPA-17 Accuracy scores that included MoCA scores and education (years) as continuous independent variables was also statistically significant [*F*(2, 256) = 57.73, *p* < 0.001, adjusted *R*^2^ = 0.31].

**FIGURE 1:**
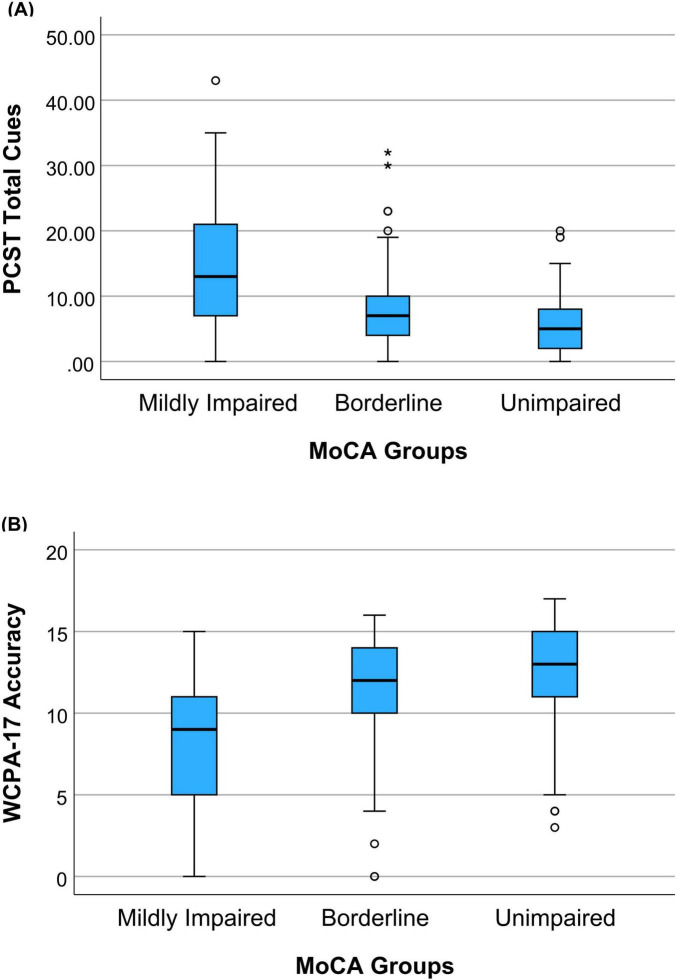
**(A)** Boxplot summary of the distribution of PCST Total Cues scores by MoCA group, **(B)** Boxplot summary of the distribution of WCPA-17 Accuracy scores by MoCA group. °Mild outlier. *Extreme outlier.

A one-way MANCOVA was performed to determine the effect of MoCA group status (mildly impaired, borderline, and unimpaired) on the two functional cognitive performance assessments, PCST Total Cues and WCPA-17 Accuracy, while controlling for education. The overall MANCOVA was statistically significant, *F*(4, 508) = 16.445, *p* < 0.001; Wilks’ λ = 0.784; η_*p*_^2^ = 0.115. Follow-up univariate ANCOVAs showed that PCST Total Cues adjusted mean score [*F*(2, 255) = 20.006, *p* < 0.001; η_*p*_^2^ = 0.136] and WCPA-17 Accuracy adjusted mean score [*F*(2, 255) = 23.216, *p* < 0.001; η_*p*_^2^ = 0.154] were statistically significantly different between the MoCA groups. Both the PCST Total Cues scores and the WCPA-17 Accuracy scores indicated lower performance for the MoCA mildly impaired group, intermediate performance for the MoCA borderline group, and the highest performance for the MoCA unimpaired group (See Table 2). Figure 1 shows a boxplot summary of the distribution of the functional cognitive assessment scores for each MoCA group.

**TABLE 2:** Adjusted means for PCST Total Cues and WCPA-17 Accuracy for each MoCA group.

Outcome	MoCA mildly impaired (*n* = 63)	MoCA borderline (*n* = 82)	MoCA unimpaired (*n* = 114)	*F*(2, 255)	*p* =	η_p_^2^
	M_adj_ (SE), 95% CI	M_adj_ (SE), 95% CI	M_adj_ (SE), 95% CI	–	–	–
PCST Total Cues	13.7 (0.8), 12.05–15.34	8.4 (0.7), 7.0–9.82	5.7 (0.6), 4.44–6.86	20.006	< 0.001	0.136
WCPA-17 Accuracy	8.4 (0.5), 7.52–9.29	11.4 (0.4), 10.62–12.12	12.2 (0.3), 11.59–12.89	23.216	< 0.001	0.154

Estimates of effect sizes were quantified via partial eta square to determine the magnitude of significant group differences and were interpreted as: 0.01 = small, 0.06 = medium, and 0.14 = large (Cohen, 1988). MoCA, Montreal Cognitive Assessment, possible range 0–30; PCST, Performance Assessment of Self-care Skills Checkbook Balancing and Shopping Task, possible range 0+; WCPA-17, Weekly Calendar Planning Activity 17-item version, possible range 0–17.

Bonferroni *post hoc* tests revealed that for PCST Total Cues adjusted mean scores, the MoCA mildly impaired group required significantly more PCST Total Cues (worse performance) than the MoCA borderline group (*p* = 0.004) or the MoCA unimpaired group (*p* < 0.001). Similarly, the MoCA borderline group required significantly more PCST Total Cues than the MoCA unimpaired group (*p* = 0.003). For WCPA-17 Accuracy adjusted mean scores, *post hoc* comparisons revealed that the MoCA mildly impaired group made more scheduling errors than the MoCA borderline group (*p* < 0.001) or the MoCA unimpaired group (*p* < 0.001). The adjusted mean differences for WCPA-17 Accuracy between the MoCA borderline group and the MoCA unimpaired group were in the expected direction but were not significant (*p* = 0.259).

## Discussion

The primary purpose of this study was to examine whether functional cognitive performance among community-dwelling older adults would differ according to their cognitive ability, represented by the tripartite grouping of the MoCA neurocognitive screening test. As hypothesized, the results show that performance on both functional cognitive assessments were significantly different based on MoCA group categorization, with better performance on the MoCA reflecting greater independence in IADL as indicated by performance on the functional cognitive measures, with medium-large effect sizes. Despite scoring in the borderline to unimpaired range on the MoCA, a subset of individuals may have difficulties with complex IADL, suggesting that the absence of impairment on the MoCA does not obviate the need for functional cognitive assessment when an individual is reporting difficulties or there are other indications of impaired performance in daily life skills (Toglia et al., 2017; Rosenblum et al., 2020; Jaywant et al., 2021). However, individuals in the MoCA borderline group scored on average, closer to the MoCA unimpaired group. We found that individuals who were assigned to the MoCA impaired group were significantly more likely to demonstrate greater difficulties with the PCST and WCPA-17. The MoCA impaired group had the fewest years of education. Nonetheless, the linear regression and MANCOVA analyses remained significant after controlling for education.

Difficulties with cognitively complex IADL are regularly present in MCI (Jekel et al., 2015; Cloutier et al., 2021). Subtle changes in the ability to perform complex IADL tasks may serve as an early warning sign and indicate need for intervention or ongoing monitoring and support to slow loss of independence (Jekel et al., 2015; Makino et al., 2020). Our findings suggest that PCST and WCPA-17 performance are reflective of subtly impaired cognitive abilities. We found significant differences in functional cognitive performance between mildly impaired and borderline and unimpaired groups on the MoCA. These results are consistent with other studies that demonstrate subtle difficulties in IADL performance (e.g., increased errors, reduced efficiency) in individuals with MCI compared to healthy adults (Giovannetti et al., 2008; Martin et al., 2019; Schmitter-Edgecombe et al., 2021). Functional cognitive assessments provide additional information regarding how an individual goes about performing complex daily activities and indicates where performance may break down. Information about how individuals’ self-correct performance after a cue on the PCST, or their ability to self-monitor and accurately schedule conflicting appointments on the WCPA-17, can guide appropriate clinical interventions. Functional cognitive assessments such as the PCST and WCPA-17 are valuable tools for the detection of cognitively mediated breakdowns in IADL function.

Our results provide evidence that a subset of individuals who score in the borderline or unimpaired range on the MoCA have functional cognitive difficulties. Both the PCST and the WCPA-17 take the form of simulated real-world activities but have distinct performance requirements. Nonetheless, these assessments appear to perform similarly in capturing subtle decrements in functional cognitive abilities not fully identified by the MoCA. As previously noted, traditional neurocognitive screening and diagnostic tests are at best, modest predictors of everyday function (Royall et al., 2007; McAlister et al., 2016; Marcotte et al., 2022). Other studies failed to find a one-to-one correspondence between neurocognitive and functional cognitive assessments (Toglia et al., 2017). The self-awareness and use of strategies assessed by functional cognitive assessments more accurately reflect the skills critical to competent everyday function. These skills are not evaluated by the MoCA or other neurocognitive screening tests (Dawson and Marcotte, 2017; Baum et al., 2023). The MoCA was never intended to assess independent community living skills that require the interplay of multiple cognitive domains. Functional cognitive assessments complement the information provided by the MoCA and other neurocognitive screening tests by requiring individuals to draw from multiple competencies during test performance resulting in outcomes that are more likely to parallel performance in daily life (American Occupational and Therapy Association, 2021). For these reasons, functional cognitive assessments contribute information distinct from cognitive screening tools such as the MoCA and therefore aid in the evaluation of real-life abilities.

This study is not without limitations. Most participants in this study were highly educated, female, and the study population was limited in racial/ethnic diversity and from one geographic region within the United States. We had an insufficient number of participants who were Black or reported other racial identities to examine subgroup differences. We did not screen for the potential impact of psychosocial factors such as depression or social isolation, which can influence independent living skills (Guo et al., 2021; Lyu et al., 2024). Additionally, social determinants of health such as income, housing, and employment status were not measured in this study but may affect the generalizability of these findings and account for some of the unexplained variance in the current analysis. Functional cognitive assessments are relatively new and the impact of these factors on test results are poorly understood. As the potential effects of psychological factors and social determinants of health have become more clearly defined in large population-based samples, we recognize that going forward it will be important to include measures of psychological and social factors into smaller studies such as ours (DeMarco et al., 2025; Ow et al., 2025). The MoCA was used to categorize cognitive ability, though more comprehensive neurocognitive testing would provide greater confidence regarding an individual’s overall cognitive status. Additional studies are needed to replicate these results in larger more diverse samples and continue to examine the relationships between cognitive and functional cognitive assessments.

Assessments that capture the everyday cognitive skills necessary to manage the complexities of real-life provide valuable information to clinicians who address everyday function of older adults. Our results found that scores on the PCST and WCPA-17 assessments are reflective of subtle differences in the cognitive abilities of older adults. The PCST and WCPA-17 directly assess functions important to everyday activity completion, though additional research is needed to determine how the scores might predict actual everyday performance in an individual’s own environment. Results suggest that relying on neurocognitive screening tests alone may not lead to a fully accurate evaluation of the capacity of older adults to successfully perform complex IADL activities. Administration of functional cognitive assessments, including the PCST, WCPA-17, or other performance-based tests (Baum et al., 2008; Wolf et al., 2017; Al-Heizan et al., 2022), present the opportunity to obtain multiple sources of information and detect more subtle differences in actual functioning (Gilmore-Bykovskyi et al., 2025).

## Data Availability

The raw data supporting the conclusions of this article will be made available by the authors, without undue reservation.
